# Using Machine Learning to Predict Remission in Patients With Major Depressive Disorder Treated With Desvenlafaxine

**DOI:** 10.1177/07067437211037141

**Published:** 2021-08-11

**Authors:** James R.A. Benoit, Serdar M. Dursun, Russell Greiner, Bo Cao, Matthew R.G. Brown, Raymond W. Lam, Andrew J. Greenshaw

**Affiliations:** 1 Faculty of Nursing, 98623 University of Alberta, Edmonton, Alberta; 2 Department of Psychiatry, 3158 University of Alberta, Edmonton, Alberta; 3 Department of Computing Science, 3158 University of Alberta, Edmonton, Alberta; 4 Department of Psychiatry, University of British Columbia, Vancouver, British Columbia

**Keywords:** antidepressants, major depressive disorder, randomized controlled trial, diagnosis, machine learning, artificial intelligence, symptom remission

## Abstract

**Background:**

Major depressive disorder (MDD) is a common and burdensome condition that has low rates of treatment success for each individual treatment. This means that many patients require several medication switches to achieve remission; selecting an effective antidepressant is typically a sequential trial-and-error process. Machine learning techniques may be able to learn models that can predict whether a specific patient will respond to a given treatment, before it is administered. This study uses baseline clinical data to create a machine-learned model that accurately predicts remission status for a patient after desvenlafaxine (DVS) treatment.

**Methods:**

We applied machine learning algorithms to data from 3,399 MDD patients (90% of the 3,776 subjects in 11 phase-III/IV clinical trials, each described using 92 features), to produce a model that uses 26 of these features to predict symptom remission, defined as an 8-week Hamilton Depression Rating Scale score of 7 or below. We evaluated that learned model on the remaining held-out 10% of the data (*n* = 377).

**Results:**

Our resulting classifier, a trained linear support vector machine, had a holdout set accuracy of 69.0%, significantly greater than the probability of classifying a patient correctly by chance. We demonstrate that this learning process is stable by repeatedly sampling part of the training dataset and running the learner on this sample, then evaluating the learned model on the held-out instances of the training set; these runs had an average accuracy of 67.0% ± 1.8%.

**Conclusions:**

Our model, based on 26 clinical features, proved sufficient to predict DVS remission significantly better than chance. This may allow more accurate use of DVS without waiting 8 weeks to determine treatment outcome, and may serve as a first step toward changing psychiatric care by incorporating clinical assistive technologies using machine-learned models.

## Abrégé

**Contexte:**

Le trouble dépressif majeur (TDM) est une affection commune et pénible qui présente de faibles taux de traitement réussi pour chaque traitement individuel. Ce qui signifie que nombre de patients nécessitent plusieurs changements de médicaments pour atteindre la rémission; sélectionner un antidépresseur efficace est typiquement un processus séquentiel par tâtonnements. Les techniques d’apprentissage machine peuvent être en mesure d’apprendre des modèles qui peuvent prédire si un patient en particulier va répondre à un traitement donné, avant qu’il ne soit administré. La présente étude utilise les données cliniques de base pour créer un modèle d’apprentissage machine qui prédit avec précision le statut de la rémission pour un patient après un traitement par desvenlafaxine (DVS).

**Méthodes:**

Nous avons appliqué des algorithmes d’apprentissage machine aux données de 3399 patients de TDM (90% des 3776 sujets de la phase 11-des essais cliniques III/IV; chacun ayant décrit utiliser 92 éléments), pour produire un modèle qui utilise 26 de ces éléments pour prédire la rémission des symptômes, définie comme étant un score de 7 ou moins maintenu durant 8 semaines à l’échelle de dépression de Hamilton (HAM-D). Nous avons évalué ce modèle d’apprentissage pour les 10 % restants des données (*n* = 377).

**Résultats:**

Le classificateur qui en a résulté, une machine à vecteur de support linéaire entraînée (SVM), avait une précision sur l’ensemble de retenue de 69.0%, significativement plus grande que la probabilité de classer un patient correctement par chance. Nous démontrons que ce processus d’apprentissage est stable en échantillonnant à répétition une partie des données de la formation et en exerçant l’apprenant sur cet échantillon, puis en évaluant le modèle appris sur les instances conservées de l’ensemble de la formation; ces mesures avaient une exactitude moyenne de 67.0% ± 1.8%.

**Conclusions:**

Notre modèle, basé sur 26 caractéristiques cliniques, s’est avéré suffisant pour prédire la rémission par DVS significativement mieux que par chance. Cela peut permettre une utilisation plus précise de la DVS sans attendre 8 semaines pour déterminer le résultat du traitement, et peut servir de première étape au changement des soins psychiatriques, en incorporant les technologies d’assistance clinique à l’aide des modèles d’apprentissage machine.

## Background

It is important for clinicians to identify the best antidepressant for patients with major depressive disorder (MDD). Selecting an antidepressant generally relies on clinical features and side-effect profile.^1^ However, meta-analyses of clinical trials for newer antidepressants found 37% of the patients do not achieve response (a relative reduction in symptoms) and 53% do not achieve remission (expressing less than an absolute threshold of symptoms) following 6 to 12 weeks of treatment.^2^ These are troubling statistics, especially as early effective treatment of depression may improve functional recovery outcomes,^3^ and each treatment failure increases the chance of overall failure and increases treatment times.^1^ Unfortunately, there are currently no reliable, well-validated tests that identify the best treatment for each patient, as we cannot accurately predict a patient's individual response, and tolerance, to any antidepressant treatment. Hence, prescribing an effective antidepressant remains a trial-and-error process.

Precision medicine attempts to identify which specific patients will respond to each specified treatment using models that can incorporate available patient information. This approach uses outcomes rather than symptom clusters to divide patients into groups, allowing for a data-driven approach. Machine learning, a subfield of artificial intelligence, includes techniques that lead to a precision medicine approach, as they can use labelled datasets listing many patients (each described by his/her specific features, together with the treatment and outcome) to produce accurate models of pharmacotherapy response. These can use potentially any type of patient information, including easily collected clinical measures (e.g. demographics, Hamilton Depression Rating Scale [HAM-D] items).^4,5^ A focus of machine learning in psychiatry has been producing models that diagnose mental health disorders, using neuroimaging data, including variants of magnetic resonance imaging (MRI; e.g. for diagnosing MDD).^6^ Using machine learning tools to predict medication efficacy using patient information would move prescribing from trial-and-error practice to more precision medicine.

To examine datasets used by other studies in this area, a review of studies using machine learning in response prediction was carried out. Of the 295 articles retrieved, we reviewed abstracts of the 167 identified as relevant. We found the most-used scales in predictive outcome assessment were the HAM-D, Montgomery-Åsberg Depression Rating Scale (MADRS),^7^ and Patient Health Questionnaire scales.^8^ We identified two groups of studies: those working with small, in-house datasets, and others using large-scale databases, such as the Sequenced Treatment Alternatives to Relieve Depression (STAR*D).^9^ Thirty-three studies (20.0%) had a sample size greater than 500. We identified a study that used data gathered during two psychotherapy-based intervention trials,^10^ but found no studies that synthesized multiple pharmaceutical clinical trials into a single dataset for building predictive models of treatment response.

The objective of this study was to develop a predictive model for treatment remission using baseline clinical information from pharmaceutical trials. This work produces an effective model by applying a machine learning technique to a large, global, multi-site dataset from 11 phase-III/IV clinical trials of desvenlafaxine succinate (DVS), a serotonin and norepinephrine reuptake inhibitor (SNRI). DVS is the primary active metabolite of the SNRI venlafaxine (thereby avoiding venlafaxine's interaction and metabolism by the liver enzyme CYP2D6), and acts as a reuptake inhibitor for both serotonin and norepinephrine with minimal effect on dopamine.^11^ We produced this model by training a learning algorithm on a subsample of the data, then evaluating the trained model on the remaining data. We then confirm the stability of that learning algorithm by running this process repeatedly across different subsamples of the dataset.

We used an approach that automatically identifies which of a set of base machine learning algorithms produces the model that is best at predicting patient response. We expanded our scope beyond a large single-country trial (e.g. STAR*D) to using a global set of instances obtained from multiple clinical trials, spanning 23 countries in five continents. We also expanded on previous methods of feature selection by applying a consistency-based feature selection method (rather than single-step feature selection methods that do not consider feature robustness as part of the selection process—for example, using Lasso or elastic net a single time on the full training set of data), to reduce the initial set of features, while demonstrating that the features picked were consistent across subsets of data.

## Methods

### Datasets

Data were obtained through a data access agreement between Pfizer Inc. and the University of Alberta. This study was approved by the University of Alberta Research Ethics Board, study Pro00064974, and all patients involved gave written consent for their anonymized data to be used. The clinical trial data included in this study were drawn from 11 DVS clinical trials. We included all available studies that had completed phase-III/IV DVS trials with adult participants, and had a HAM-D outcome measure.^5^

Our dataset combined data from 11 phase-III/IV DVS clinical trials carried out between 2003 and 2011, with a total enrollment of 7,051 patients. Table 1 provides the clinical trial characteristics and proportion of subjects from each clinical trial included in results. Our inclusion criteria (given below) reduced this to 3,776 patients, from which we randomly selected a training set of 3,399 patients (90%), leaving a holdout set of 377 patients—held aside from the machine learning process, see Table 2.

**Table 1.** Clinical Trial Characteristics and Enrollment.

Dataset	Year	Locations	Enrollment	% Included	Remission rate (of included)
NCT00277823	2006–2007	United States	480	55.21	41.2
NCT00300378	2006–2007	Croatia, Estonia, Finland, France, Latvia, Lithuania, Poland, Romania, Slovakia, South Africa	480	66.25	35.1
NCT00369343	2006–2008	United States	381	61.42	40.6
NCT00384033	2006–2007	United States	638	42.32	26.3
NCT00406640	2006–2008	Argentina, Chile, Colombia, Mexico, Peru, United States	595	44.54	43.7
NCT00445679	2007–2009	China, India, Republic of Korea, Taiwan	807	67.29	45.9
NCT00798707	2008–2010	Japan, United States	709	62.20	20.8
NCT00824291	2009	United States, Canada	437	55.38	38
NCT00863798	2009–2010	United States	682	63.64	20.3
NCT01121484	2010–2011	United States	439	42.60	23.5
NCT01309542^a^	2003–2006	Estonia, Finland, Former Serbia and Montenegro, France, Germany, Latvia, Lithuania, Poland, Slovakia, South Africa, United States	1403	41.13	56.7

^a^ Open-label trial.

**Table 2.** Dataset Statistics: Mean Demographic Information and HAM-D Scores for Training and Holdout Sets.

	Training	Holdout
*n*	3399	377
Age (years)	44.0	43.6
Sex (% female)	69.8	67.9
Ethnicity (% White)	65.1	65.0
HAM-D baseline	21.3	21.3
HAM-D week 8	10.9	11.3
Remission rate %	37.9	37.9

### Inclusion Criteria

Within each study, we only included patients with a primary diagnosis of MDD, treatment in a DVS monotherapy arm of a trial, and completion of a 17-item HAM-D assessment at both baseline and 8 weeks. Data from patients who did not meet all of these inclusion criteria were not included in model training or analysis. We did not consider other comorbidities: trial participation required subjects be otherwise healthy, with a primary MDD diagnosis.

### Outcome Measures

We assessed treatment outcomes according to the clinician-reported 17-item HAM-D, obtained 8 weeks after the start of the trial, with the key outcome symptom of remission defined as a HAM-D score of 7 or less at endpoint.^12^

### Features Considered

Our training data included 92 features, whose values were known for each patient at the start of the trial. These were composed of psychiatric scale items, that is, individual items from the Clinical Global Impressions Scale (CGI),^13^ MADRS,^7^ and HAM-D, demographic data (e.g., age and ethnicity), lab tests (e.g., free T4 and white blood cell count), and a measure of polypharmacy. The polypharmacy measure is a simple count of the number of pills taken each day by the patient, which includes adjunct medications and nonprescription drugs and supplements, based on the patient's self-report question at baseline: “How many different other pills do you take each day?”

We filled missing data points (e.g., the age of a patient who was missing the value for age), using mean imputation (i.e., replacing each missing value with the mean of that feature's nonmissing values). This required imputing 0.284% of all data, with a maximum imputation of 8.87% for any single feature (free T4); note all values were present (over all 3776 patients) for 62/92 features.

### Predictive Model

**Figure 1.**
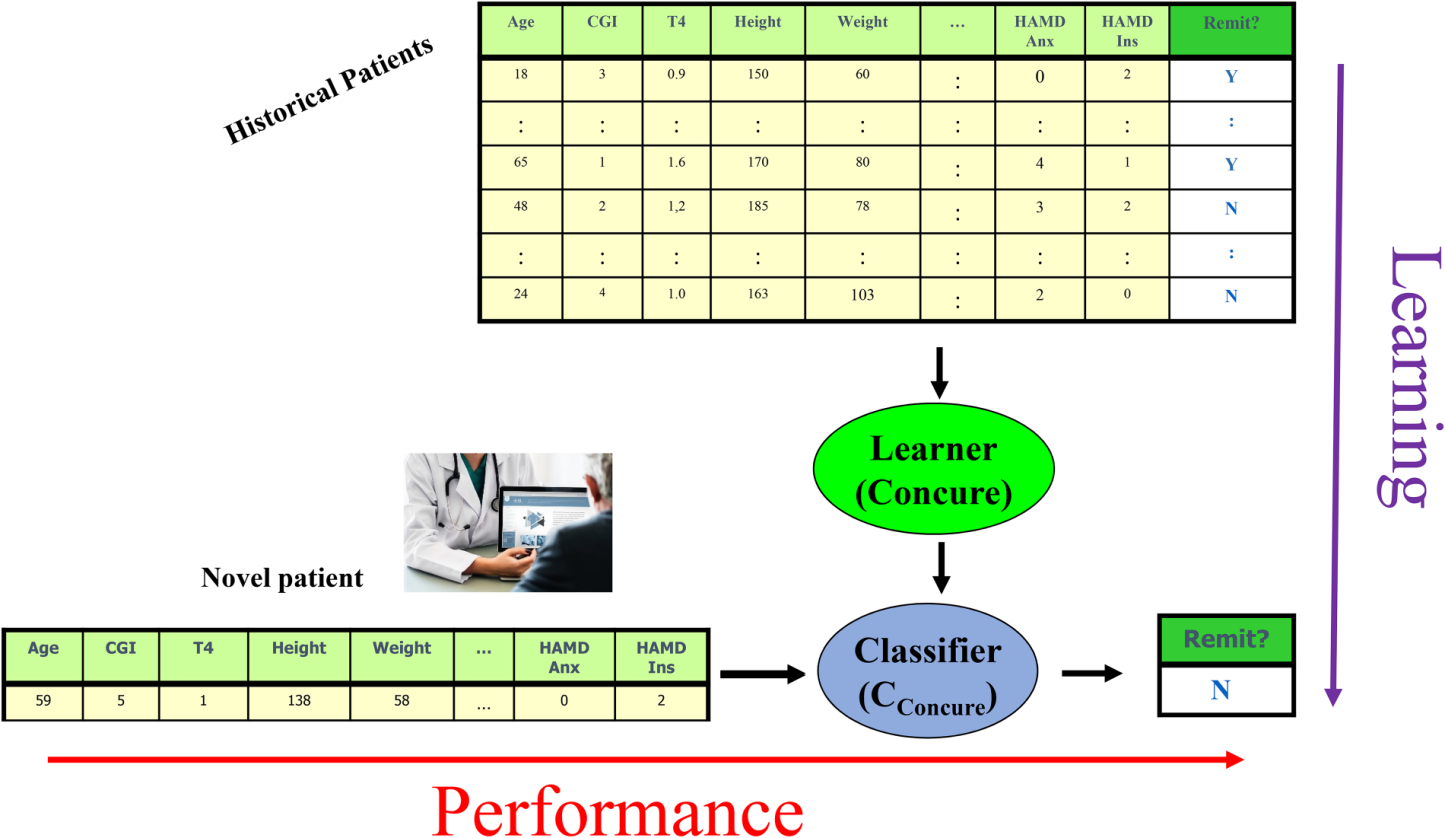
Distinguishing between the learning algorithm (top to bottom) that trained on historical data to produce a classifier; and the performance phase (left to right), that runs that trained classifier on a novel patient, to produce a prediction.

The machine learning algorithm was designed in Python 3, primarily using the pandas and sklearn libraries for preprocessing and modeling, respectively. We applied this machine learning algorithm to a labelled training dataset, which describes each patient as the values of a set of clinical features, taken when that patient entered the clinical trial. We use supervised machine learning, since we have labelled data that provide the specified outcome (remission). There would be little value in applying unsupervised machine learning to this problem, beyond creation of additional features, since the data explicitly provides the labels (remitters vs. nonremitters). Each patient has a label indicating whether that patient remitted at 8 weeks, indicated by a HAM-D score of 7 or less. Running the learning algorithm on the training data produced a trained classifier, which can predict whether a novel patient (a person who was not in the training set) would experience symptom remission at 8 weeks, based on his/her personal features (psychiatric scale scores, etc.; Figure 1).

The learning algorithm involves three sequential steps (each described in detail below):

1. Identify the subset of features that appear most informative for predicting remission versus nonremission.
2. Identify the best “base learner,” using this subset of features.
3. Run the best base learner on the dataset projected onto the feature subset, to produce a classifier.

To motivate the first step, “feature selection”: It is well known that models trained with too many features often overfit; this has led to many standard methods that select a smaller set of features. Here, our learning algorithm initially partitions the input dataset into five disjoint subsets, called “folds,” balanced to the proportion of remitters versus nonremitters. The algorithm then selects features from each training fold using Lasso (a regularization method that effectively removes less useful features from the model), then takes common features between these folds to form a feature subset. This produced the set of 26 features, shown later. It then applies 11 algorithms, each of which produces a classifier from a training dataset (using five-fold internal cross-validation), and found that a linear support vector machine (SVM) classifier had the highest accuracy (i.e., the percentage of correctly classified instances). It then ran this linear SVM learner on all the labelled training data, using the selected subset of features, to produce a final trained classifier (see Supplemental Material for details of this process). This process—of training different base algorithms on a subset of data, then evaluating the trained classifier on the held-out set—allows selection of which base learner is best, in a statistically fair and appropriate fashion.

We estimate the trained algorithm's predictive accuracy in two ways. We chose accuracy (see Supplemental Material for equation) as our performance measure as it equally weights type I and II error. Firstly, we use (external) cross-validation over the training data: here, we run the entire learning process (including the feature selection) 5 times,^14^ each time on 80% of the training data, and evaluate that classifier on the remaining 20% of training data. As we evaluated each learned model on instances that it was not trained on, the estimate is not optimistically biased. We then report the average accuracy of these five performance evaluations as an estimate of the accuracy on the overall learned model (the one based on all of the data; the Supplemental Material provide a formal description of this process).

Secondly, we applied the trained classifier to our held-out patient dataset to determine whether this model generalizes to novel patients from data that were entirely separate from the dataset used that was used to train the classifier. This returned a single accuracy value. To assess confidence that the accuracy value produced was above chance (i.e., our algorithm learned something useful in the data), we used bootstrapping, based on 10,000 draws with replacement of size *n* = 377 from the holdout set—considering how many of these 377 instances had the correct label (from our learned model). The confidence level that our trained classifier performed significantly above chance was determined by computing whether (at least) 95% of these 10,000 accuracy values were above the chance probability of correctly classifying a patient by assuming that all patients were nonremitters (corresponding to the majority class of patients here, at 62.1%).

## Results

### Feature Selection

Our algorithm found the following 26 features were selected by all five folds (grouped by feature type):

1. Nine countries of origin (with each country considered as an individual binary feature): Argentina, Canada, China, Colombia, Croatia, Finland, Japan, Mexico, and the USA.
2. One ethnicity (American Indian/Alaska Native).
3. Eight HAM-D Scale items:
  - anxiety/somatic (anxiety concomitants, e.g., headaches, sweating);
  - feelings of guilt (including rumination, delusions, hallucinations of guilt);
  - genital symptoms (libido);
  - loss of insight;
  - insomnia/early (difficulty falling asleep);
  - somatic symptoms/gastrointestinal;
  - somatic symptoms/general (e.g., muscle ache, loss of energy, fatigability);
  - work and activities (e.g., difficulty working or doing hobbies, being productive).
4. Three MADRS Scale items:
  - apparent sadness,
  - pessimistic thoughts,
  - reported sadness.
5. One measure of polypharmacy (pill count including supplements, nonprescription drugs).
6. Four lab tests:
  - albumin,
  - creatinine,
  - potassium,
  - urine pH.

Please note it is unlikely that highly correlated features would be selected together for the model, as multicollinearity adds unnecessary complexity without a corresponding increase in predictive value to justify that feature's inclusion.

### Classifier Selection

The classifier learned by the SVM base learner was consistently the most accurate of the 11 trained classifiers tested, in the internal cross-validation folds. This classifier considers the 26 selected features in 26-dimensional space and generates a hyperplane (i.e., a plane in more than three dimensions) that best separates the two classes; we then classify a novel patient based on which side of the hyperplane its 26D feature tuple appears.

### Estimating the Quality of the Learning Algorithm

We evaluated results in two ways. First, five-fold cross-validation accuracy (with respect to the training set) was 67.0% ± 1.8%. A two-tailed *t*-test shows this is significantly different from the 62.1% chance accuracy, *P* = 0.0065.

### Classifier Validation and Generalizability

**Figure 2.**
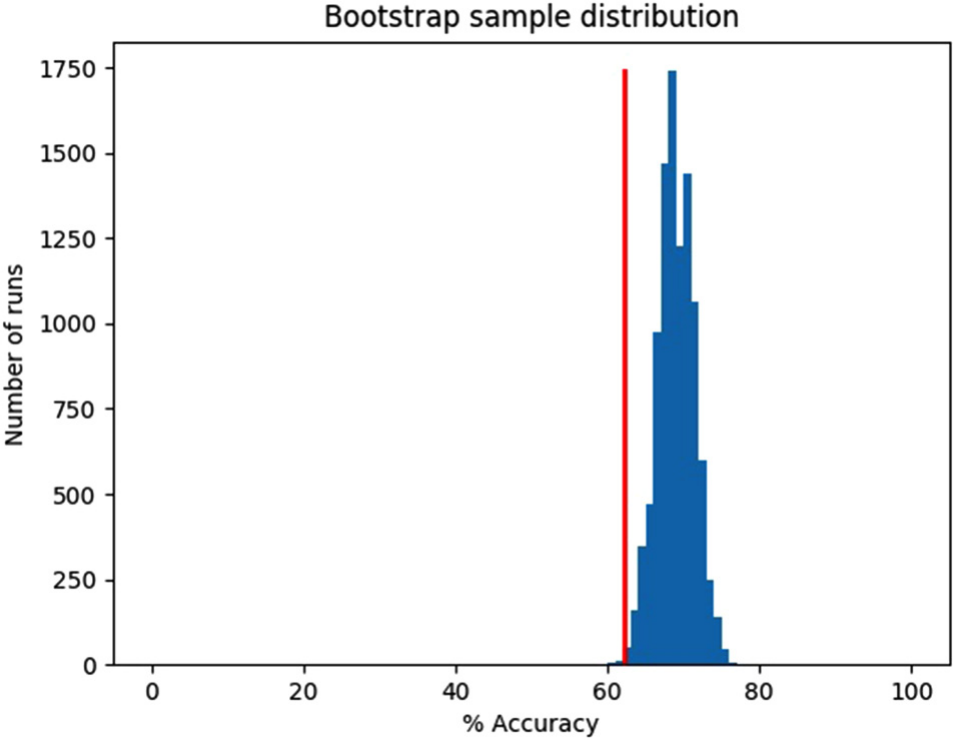
Holdout data bootstrap, mean accuracy = 69.0%, chance accuracy = 62.1%, 10,000 samples with *n* = 377 instances/sample, 0.25% of runs below chance.

Second, to explore the external generalizability of our learned 26-item model, we tested it on holdout data (*n* = 377, 37.9% remitters, 62.1% nonremitters). Its mean accuracy was 69.0%. Figure 2 shows that the bootstrap sampling fell below chance accuracy 0.25% of the time, indicating we can be confident that the algorithm is learning to classify remitters from nonremitters at above-chance levels.

## Discussion

Our learning algorithm produced a trained classifier capable of identifying, with modestly better-than-chance performance, whether new patients diagnosed with MDD will experience symptom remission after 8 weeks of DVS monotherapy. This classifier demonstrates a simple model, using 26 easily obtained clinical features at baseline, can predict symptom remission at better than chance levels, even when applied to holdout data not used to train the classifier.

For comparison with other studies, Chekroud et al.^4^ trained a machine-learning model using a large citalopram trial. Even though their model was predicting response to a different antidepressant, citalopram, our models shared three HAM-D features: loss of insight, somatic anxiety, and somatic energy. Interestingly, if we split HAM-D items into four previously proposed symptom clusters based on principal component analysis (mood, sleep/psychic anxiety, weight/somatic anxiety, and insight/appetite),^15^ both models contain HAM-D items from all four clusters. This may suggest that predicting treatment outcomes will be strongest in models that capture and consider multiple MDD subtypes.

Comparing our model to Iniesta et al.^16^ combined outcome prediction model for escitalopram and nortriptyline, which integrated demographic with clinical variables to train an elastic net-based predictor of treatment outcome, our model shared MADRS apparent sadness and HAM-D work and activities. Apparent sadness relates to a core feature of MDD (mood) and was therefore an expected feature and workplace functioning has previously been shown to be improved by both DVS and escitalopram.^17^

Recall that our machine learning approach did not start with predefined features to include, and so it chose a set of features—which we see includes both expected and unexpected features. Of the 26 features selected, 11 were items from well-validated psychiatric scales: eight based on HAM-D items, and three on MADRS items. It also included nine countries of origin, one ethnicity feature, four lab tests, and one measure of polypharmacy. Inclusion of the HAM-D “loss of insight” item was unexpected, as it is the least frequently occurring symptom of depression at baseline, and shows the least change of any item at treatment termination.^18^ However, this result is consistent with the findings of Chekroud et al.,^4^ suggesting the machine learning algorithm finds value in using this feature. The polypharmacy feature was also used as a predictor of DVS efficacy, even though the simple nature of the item (number of pills taken daily by the patient) does not allow a fine-grained model for each adjunct medication. As there were 1,507 different medications and other supplements listed that varied across patients, adding each one to the model (rather than combined into a single feature) would likely increase the risk of overfitting due to creating a large, sparse input matrix. The inclusion of country of origin is supported by a recent study examining the treatment effect of an antidepressant between different countries: mean duloxetine response was negatively associated with gross national income.^19^

Since learning algorithms such as SVM use multivariate combinations of features, interpreting the individual contributions of each feature to the model (both as a weight and in the context of other features present) can be misleading. However, the features used by our trained classifier will be useful in real-world deployments, as they are easily captured, even in low-income jurisdictions and marginalized populations that have limited or no access to advanced medical technology such as MRI. This perspective is strengthened by the international datasets used, suggesting that future trained classifiers have potential to be deployed globally in clinical settings.

We had a choice of using MADRS, CGI, or HAM-D scales to assess patient outcome. We selected the HAM-D since it has been the most widely used standard for 40 + years of MDD research,^20^ and is one of the three FDA-accepted endpoints for assessing antidepressant efficacy.^21^ We hope to extend this research in future studies in two ways. Firstly, testing scale-based outcomes against patient self-assessments of remission allow us to create a better proxy measure conducive to predictive modeling. Secondly, using a combined label for symptomatic and functional assessment (e.g., HAM-D and Sheehan Disability Scale) incorporates both functional and symptomatic remission, giving a more complete picture of whether a patient responded to treatment.^22^

While the features selected have proven to be sufficient for significantly above-chance predictions, this analysis does not show them to be causally related to remission of depression.^23^ This is a good start toward using easily obtainable features to predict remission, but better predictive accuracy is required to justify clinical use. While the literature has described associations between these features and depression treatment response, a different learning process (on this or a similar dataset) might select an entirely different subset of features. That is not to say the features selected are irrelevant: given novel patient data, the analysis suggests that our trained classifier should accurately predict remission at above-chance levels.

We addressed missing data in a very simple way: mean imputation.^1^ We did try an alternative strategy, median imputation (i.e., replacing each missing value with the median of that feature's nonmissing values), but found this preprocessing step led to classifiers that were less accurate. In some cases, we did not include features if they were missing from some trial datasets; these were excluded during data preprocessing (e.g., body mass index [BMI], a factor found to be important for treatment response prediction).^16^ Our results could have benefitted from more modalities of data, as other studies have shown that these pooled models sometimes outperform models with fewer data types.^17^

In summary, this machine learning approach is an important step forward for clinical practice, because it demonstrates the feasibility of using easily collected baseline data to improve prediction of antidepressant efficacy. More research is required to improve and replicate prediction accuracy of machine learning algorithms before they can be applied in clinical care. However, applied broadly, machine-learned models of treatment prediction may change clinical practice in two ways. Firstly, classification models (such as the one in this study) can identify which patients should (not) receive this treatment. Secondly, machine learning regression models may allow clinicians to compare remission probabilities of many drugs, toward identifying the best class of drugs (or the best for a given cost, in terms of dollars, or for side effects). These two advantages will help clinicians target both a class of drugs and an individual drug, for each individual patient, based on that patient's characteristics.

## Limitations

Datasets from clinical trials are often different from a real-world setting. Exclusion criteria used by randomised control trials (RCTs) limit recruitment to a study population with fewer confounds than are found in real-world settings (e.g., more comorbidities and complex cases). Models learned from these data may not account for variability associated with patients excluded from the RCT. For example, respondents to RCT recruitment have been shown to differ significantly from nonrespondents in demographics, socioeconomic status (SES), and behavioral characteristics.^24^ Testing our algorithm in a live clinical setting will address this issue empirically by measuring its effectiveness directly on the patient population of interest.

We found that the majority of patient cases we excluded during data cleaning prior to model creation and analysis lacked a week-8 HAM-D score. Increasing the included cases by changing this outcome measure to a different treatment duration would have required assessing remission at a nonstandard treatment duration, while using a technique such as imputation to fill missing values would have resulted in a less meaningful set of data. Separate from excluded patient cases, missing input feature data may have resulted in a feature (e.g., free T4) being ignored during the algorithm's feature selection step due to its lower variance resulting from mean imputation to fill missing values.

## Supplemental Material

sj-docx-1-cpa-10.1177_07067437211037141 - Supplemental material for Using Machine Learning to Predict Remission in Patients With Major Depressive Disorder Treated With Desvenlafaxine Click here for additional data file. Supplemental material, sj-docx-1-cpa-10.1177_07067437211037141 for Using Machine Learning to Predict Remission in Patients With Major Depressive Disorder Treated With Desvenlafaxine by James R.A. Benoit, Serdar M. Dursun, Russell Greiner, Bo Cao, Matthew R.G. Brown, Raymond W. Lam and Andrew J. Greenshaw in The Canadian Journal of Psychiatry

## Notes

1. Although it is well known that mean imputation reduces the standard deviation of data, our focus was just the accuracy of the learned classifier (on held-out data); the standard deviation of a feature, or of the data, was not by itself relevant.
