# Supplementary material for: Using Machine Learning to Predict Remission in Patients With Major Depressive Disorder Treated With Desvenlafaxine
Source: Can J Psychiatry. 2021 Aug 11;67(1):39–47. doi: 10.1177/07067437211037141 (PMC8808003; doi:10.1177/07067437211037141)
Supplement: sj-docx-1-cpa-10.1177_07067437211037141 - Supplemental material for Using Machine Learning to Predict Remission in Patients With Major Depressive Disorder Treated With Desvenlafaxine [file sj-docx-1-cpa-10.1177_07067437211037141.docx]

## Supplementary Materials

Table S1. List of 92 features included in the training dataset

| **Lab Tests** | **HAM-D** | **Demographics** | |
| --- | --- | --- | --- |
| Albumin | Agitation | Age | |
| Alkaline Phosphatase | Anxiety/Psychic | Sex | |
| Basophils | Anxiety/Somatic | Ethnicity | |
| Bilirubin | Depressed mood | American Indian or Alaska Native | |
| Chloride | Feelings of guilt | Asian | |
| Cholesterol | Genital symptoms | Black or African American | |
| Creatinine | Hypochondriasis | Hispanic or Latino | |
| Eosinophils | Insight | Middle Eastern or North African | |
| Free T4Z | Insomnia/Early | Native Hawaiian or Other Pacific Islander | |
| Gamma-glutamyl transferase | Insomnia/Middle | Other | |
| Glucose | Insomnia/Late | White | |
| HDL Cholesterol | Loss of weight | Study Location | |
| Hematocrit | Retardation | Argentina | Poland |
| Hemoglobin | Somatic symptoms/ Gastrointestinal | Canada | Romania |
| LDL Cholesterol | Somatic symptoms/ General | Chile | Slovakia |
| Lymphocytes | Suicide | China | Taiwan |
| Monocytes | Work and Activities | Colombia | United States |
| Neutrophils | **MADRS** | Germany | Yugoslavia |
| Platelet count | Apparent sadness | Estonia | South Africa |
| Potassium | Concentration difficulties | Finland |  |
| Protein | Inability to feel | France |  |
| Red blood cell count | Inner tension | Croatia |  |
| SGOT (aspartate aminotransferase) | Lassitude | India |  |
| SGPT (alanine aminotransferase) | Pessimistic thoughts | Japan |  |
| Sodium | Reduced appetite | Korea |  |
| Triglycerides | Reduced sleep | Lithuania |  |
| Uric acid | Reported sadness | Latvia |  |
| Urine pH | Suicidal thoughts | Mexico |  |
| Urine specific gravity | **CGI** | **Other** | |
| White blood cell count | Severity | Polypharmacy count* | |

*Count of each reported prescription/non-prescription medication and supplement taken at baseline

#### Details of the Concure learning algorithm:

Concure (so named because it relies on the CONcurrence of features across subsets to predict a CURE as defined by remission) takes an input a labeled dataset D (each row describing a patient, and each column a component feature of the patient’s description, along with the outcome of Remit or No Remit), and returns a classifier C_Concure_ = Concure(D); that classifier, in turn, takes a description of a patient, and returns the remission label.

First, Concure needs to determine the appropriate subset of features to include. To do this, Concure partitions the labeled training dataset D into 5 disjoint sets of patients $D = D_{1}\cup D_{2} \cup\cdots\cup D_{5}$, and sets $D_{-j} = D - D_{j}$. For each i, Concure fits a Lasso (Least Absolute Shrinkage and Selection Operator) model with Lars (Least Angle Regression) using AIC (Akaike Information Criterion) to D_-j_. This produces 5 classifiers, each using its own set of features -- here 5 feature subsets ${{\{ f}_{1,}..., f_{5} \}}$. Concure computes the intersection of the features of these sets to produce a set of common features, $f^{*}=f_{1} \cap f_{2}... \cap f_{5}$, and then focuses on just these common features within D, which we call D[$f^{*}$].

Next, Concure wants to identify a good base learner BL*. It considers the following 11 base learners {BL_i_ } (more information on each learner can be found on the scikit-learn website, scikit-learn.org):

7 stand-alone learners:

- random forest (max tree depth= 5, no bootstrapping, max # features= # input features)
- extra trees (aka **ext**remely **ra**ndomized trees; max tree depth= 5, no bootstrapping, max # features= # input features)
- k nearest neighbors (neighbors = # input features, uses manhattan distance for the Minkowski metric)
- naive bayes (default parameters)
- decision tree (max tree depth= 5, no bootstrapping, max # features= # input features, uses information gain criteria for measuring split quality)
- (linear) support vector machine (L1 penalty)
- neural net (initial learning rate= 0.0001, 500 iterations max, 3 hidden layers, each of size = ⅔ * # input features)

3 meta-learners that combine a stand-alone learner in various ways:

- gradient boosting (loss set to ‘exponential’: recovers AdaBoost algorithm, 2 nodes per tree)
- adaboost (default parameters)
- bagging (random forest base learner)

A voting learner (MM) is also considered that first trains the three individual meta-classifiers shown above, then returns a single trained classifier C_MM_. When given a novel patient, C_MM_ returns a label based on the label and confidence in that label given by its 3 trained (meta-) classifiers.

To determine the best base learner, Concure needs to estimate the quality of each base learner BL_i_(.). Here, we would like to first run each such learner on the full dataset D, and then evaluate that learned classifier on another test dataset, from the same “target distribution” -- the one that gave rise to D. However, we cannot use D as the test dataset, as the target should be disjoint from the training dataset. So, instead, Concure estimates the quality of applying the base learner BL_i_(.) to D, by instead running BL_i_(.) on D’, where D’ is similar to D, then evaluating the resulting base classifier, BC_i_ = BL_i_(D’) , on a set D” that is similar to D, but (importantly) is disjoint from D’.


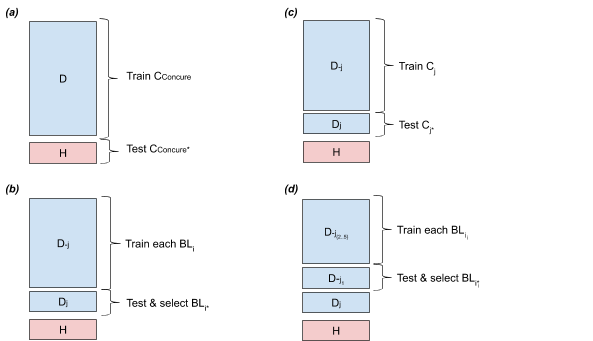


Figure S1. Data splitting process for C_Concure_

Figure A1 shows the data splitting process for (a) ${C_{Concure}}($training & evaluation of a final trained classifier  ${C_{{Concure}^{*}})}$, (b) $BL_{i^{*}}$selection to determine a base learner to be used in $C_{Concure}$, (c) Training of one of five ${C_{j^{*}}}$to provide a meaningful estimate of $C_{Concure}$ quality, and (d) $BL_{{i_{j}}^{*}}$selection to determine which base learner will be used for $C_{j}$.

Here, we use 5-fold cross-validation, using the same { D_i_ } shown above (but here, using only the $f^{*}$ features for each). For each j = 1..5, Concure runs each of 11 base learners on 4 of the 5 subsets $D_{-j} = D - D_{j}$; this produces 11 classifiers trained on the $f{}^{*}$projection of $D_{-j}$-- one for each base learner -- call each $BC_{i,-j}(.)$for the base learner BL_i_(.). It then runs each on the “held-out’ fifth subset, $D_{j}$ -- and uses the results to compute its empirical accuracy:

$a_{i,j} = acc( BC_{i,j}(.), D_{j} ) = \frac{1}{|D_{j}|}\sum_{(x,y) \in D_{j}} I[ y == BC_{i,j}( x ) ]$

where $I[ y == BC_{i,j}( x ) ]$ is 1 if $y$ is equal to $BC_{i,j}( x )$, and is 0 otherwise.

The cross-validation process actually runs this “train on ⅘, then test on remaining ⅕” process five times, each time holding-out one of the 5 folds. This produces 5 values $\{ a_{i,1}, a_{i,2}, ..., a_{i,5} \}$, for each base learner $BL_{i}$. Concure then computes the average for each $BL_{i}$:

$s(BL_{i}) = \frac{1}{5}\sum_{j=1}^{5} a_{i,j}$

then claims the best base learner is the one with the highest score $j^{*}= argmax_{i}\{ s(BL_{j} ) \}$ . Here, Concure(.) found that the SVM base learner had the highest accuracy.

Given this, Concure then ran the best base-learner $BL_{j^{*}}=$ SVM, on $D[f^{*}]$ (the 26 feature $f^{*}$ projection of the entire dataset D), to produce our trained classifier ${C_{Concure}}(.)$.

#### Estimating Predictive Accuracy of Concure Classifier (${\boldsymbol{C}_{\boldsymbol{Concure}}}$)

We now want to estimate the predictive accuracy of this resulting ${C_{Concure}}(.).$ We obtain this estimate by running 5-fold cross -validation:

Here, we again divided $D$ into 5 disjoint sets of patients, but now, we ran the entire Concure(.) process on each subset $D_{-j}$: Concure partitions each $D_{-j}$ into 5 partitions, uses those partitions to find the best feature set ${f^{*}}_{j}$, then uses $D_{-j}[ {f^{*}}_{j}]$ to find the best base learner, indexed by ${{i_{j}}^{*}}$, then runs this base learner $BL_{{i_{j}}^{*}}$on $D_{-j}[ {f^{*}}_{j}]$ to produce a classifier, here $C_{j} =$Concure( $D_{-j}$). We then run each of these $C_{j}$’s on the associated held-out $D_{j}$’s, to find its accuracy. We then return the average of these accuracies, as our estimate of the quality of Concure( D )’s C_Concure_ .

N.b., the 5 feature sets $\{ {f^{*}}_{1}, ..., {f^{*}}_{5} \}$may be different for different $j$’s, (eg, ${f^{*}}_{1}$ may be different from ${f^{*}}_{3}$) and from Concure(D)’s 26-feature ${f^{*}}$, the various base learners selected $\{BL_{1_{j^{*}}}, ..., BL_{5_{j^{*}}}\}$ may be different from each other, and from Concure(D)’s base learner $BL_{j^{*}}$= SVM, and the classifiers ${\{C}_{1}, ..., C_{5}\}$ may be different from one another, and from Concure( D )’s C_Concure_.

This is irrelevant -- the only reason to generate these 5 $C_{j}(.)$ classifiers, and then evaluate them on their respective held-out subsets, is just to produce the 5 accuracy values, whose average is used as an estimate of the quality of running Concure on D.
